# Laparoscopic-assisted excision of a huge polycystic omental lymphangioma in a 3 year old patient presenting with acute abdomen: case report and review

**DOI:** 10.11604/pamj.2021.38.228.26607

**Published:** 2021-03-01

**Authors:** Maria Tsopozidi, Chrysostomos Kepertis, Dimitrios Godosis, Vasilios Mouravas, Charikleia Demiri, Ioannis Spyridakis

**Affiliations:** 1 Second Pediatric Surgery Department, Aristotle University Thessaloniki, General Hospital Papageorgiou, Thessaloniki 54640, Greece

**Keywords:** Lymphangioma, greater omentum, acute abdomen, laparoscopic management, case report

## Abstract

Lymphangioma is a rare benign neoplasm affecting mainly children. In this report we present a complicated case of polycystic omental lymphangioma in a 3 year old female presenting with acute abdomen. The patient underwent a laparoscopic-assisted excision of the lesion and had an excellent postoperative course. We discuss the effectiveness and advantages of this laparoscopic surgical approach in children and elaborate on the current literature.

## Introduction

Lymphangiomas are rare congenital malformations of the lymphatic system, both of childhood and adulthood, with a prevalence of around 1, 1: 10,000 to 5, 3: 10,000 live births [1]. The majority of cystic lymphangiomas are seen in the neck at about 70% to 80% and the rest 20% to 30% in the axilla, abdominopelvic cavity, extremities, trunk, and thorax [1]. Abdominal lymphangiomas represent an even smaller percent of this condition accounting for about 3% to 9.2% [2]. Namely a relatively recent study [3] demonstrated that 25% are usually located in the mesentery, 40% in the omentum and 35% in the retroperitoneum.

Most cases of lymphangioma are asymptomatic. However, probably owing to a smaller abdominal cavity, children more than often develop symptoms [2]. Little is known about omental lymphangioma, except for its rareness and occasional abrupt presentation often mimicking other more obvious causes of acute abdomen such as peritonitis from a perforated appendicitis or ovarian torsion. Usually, emergency surgical management is required, though on occasions elective surgery seems also a possibility [2]. Nonetheless, even as incidental findings they require excision shortly after the diagnosis is made, owing to issues concerning resectability and high rate of complications [4]. Herein, is described the case of 3 year old female with acute abdomen due to omental lymphangioma.

## Patient and observation

A 3 years old female was transferred to our Emergency Department due to acute abdominal pain, for further investigation and potential surgical management, after a brief hospitalization in the Pediatric Department of a peripheral hospital. The child suffered from a gradually intensifying abdominal pain which began two days before presentation, accompanied by low grade fever appearing later during the course of the illness. On arrival, vital signs were stable, within normal limits and the clinical examination revealed tenderness to palpation, predominantly in the right lower quadrant, and a distended abdomen with abdominal guarding suggestive of peritonitis due to appendiceal perforation. According to the history of the present illness no other complaints indicative of gastrointestinal discomfort (e.g. vomiting, diarrhea) were reported.

**Figure 1:**
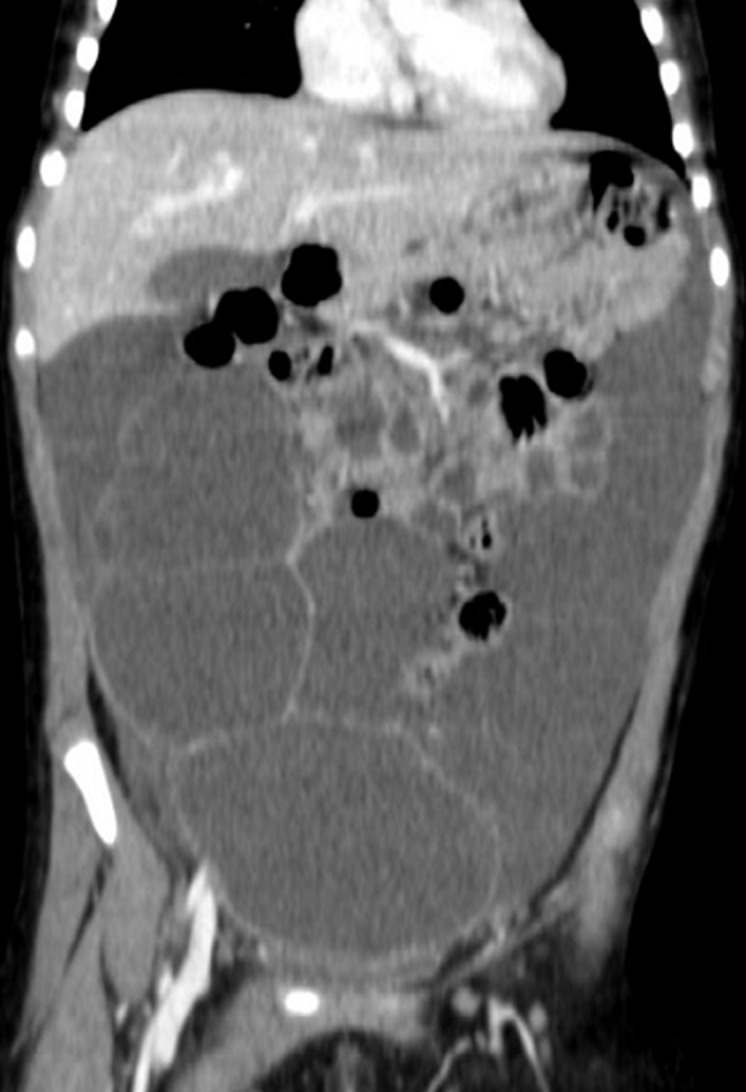
computed tomography image demonstrating a giant multilocular cystic lesion, occupying the entire abdominal cavity laterally

**Figure 2:**
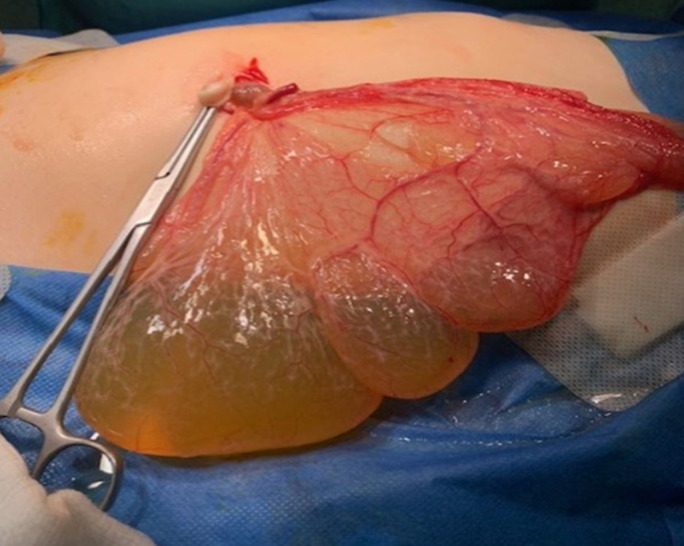
image of the cystic lesions of the omentum externalized from the umbilical incision

A previously performed sonographic imaging showed a cystic lesion measuring up to 14.5cm, presumably arising from the left ovary. The white blood count on admission was 23,000/mm^3^ with high levels of neutrophils. In this context a computed tomography (CT) scan of the abdomen was performed that demonstrated a giant multilocular cystic lesion occupying the entire abdominal cavity laterally, dislocating proximally the intestinal loops, measuring up to 20cm in length and 15cm in width (Figure 1). Laparoscopic exploration revealed polycystic configuration of the greater omentum, over its entire length, along with mild adhesions and yellowish serous fluid accumulation both inside the cysts and in the lower abdomen, with no other pathology of the abdominal cavity. The entire omentum was externalized via the umbilical incision and gradually resected in parts (Figure 2). The postoperative course was satisfactory. The child had an uneventful follow-up, being discharged five days postoperatively, with no signs of recurrence 6 months later. The histopathological report confirmed the diagnosis of cystic omental lymphangioma.

## Discussion

Lymphangiomas are rare benign amartomatous malformations of the lymphatic tissue. They are considered congenital lesions with up to two thirds being noted at birth and 95% presenting before two years of age [5]. Lymphangiomas occur primarily on the cervix and axilla and secondarily on other areas of the body. Though the pathogenesis of lymphangiomas remains an equivocal and complex matter, they are thought to occur due to sequestration of lymphatic tissue that fails to connect with the lymphatic system during embryonic development [6].

They can be classified into three types: 1) capillary; 2) cavernous and 3) cystic, which is also known as cystic hygroma [5]. Abdominal lymphangiomas are mostly cystic, but can be cavernous [7], comprising less than 5% of all lymphangiomas in children [3]. The incidence of mesenteric and omental cysts is around 1 in 20,000 children [4], with the latter accounting for up to 2.2%. They have clinical importance owing to the danger of serious complications arising, such as enlargement, intestinal obstruction, bleeding, torsion, infection or rupture [2]. In our case the patient presented with an acute abdomen due to mass effect and rupture of the cysts, possibly after acute enlargement of the lesion. Straw-colored fluid accumulation in the lower abdomen, accompanied by adhesions is likely indications of the aforementioned course of events.

Omental lymphangiomas are rare abdominal malformations that due to the capacity of the abdominal cavity to expand do not develop symptoms early, unless complications arise [7]. Ultrasonography combined with an abdominal CT scan lead to a diagnostic accuracy of up to 90% according to Li *et al*. [7]. The same diagnostic approach was also followed in our case, though a definite diagnosis was possible only after the laparoscopic exploration due to the atypical symptoms and imaging resemblances with other conditions (e.g. ovarian cysts, cystic renal tumors and others). Most of these cases require immediate surgical management, though elective surgery is possible under certain conditions, for a better evaluation of the cystic mass and complete resection is mandatory, though not always feasible [2].

Surgical management consists usually of an open surgical exploration and excision of the lesion, with laparoscopic management gaining popularity over the years. According to two case series studies in children [8, 9], complete laparoscopic excision of omental lymphangiomas presents no special challenges. Even in emergency situations with challenging huge masses, like in our patient, a laparoscopic-assisted excision may be the appropriate mode of treatment, yielding excellent results. A similar case of an 8 years old premenarchal girl with a huge omental lymphangioma, which was published by Takeda *et al*. in 2017, was managed the same way with equivalent results and no recurrence after five years [10].

Lymphangiomas generally are known to have high recurrence rates after excision. In a case series study, a postoperative recurrence rate of 10% was noted, probably due to incomplete resection [7], while lymphangiomas have an aggressive biological behavior often invading adjacent organs and structures, contradicting their otherwise benign nature [5]. Consequently, in some cases, segments of nearby tissues (e.g. bowel) might be necessarily excised to avoid recurrence, except for cases when prohibitive factors coexist, such as great proximity to vital organs or infiltration of large segments of vital organs that might compromise function [4, 7]. Nevertheless, the reported success rate for complete excision is from 82% to 95% for abdominal lesions and only 55% for retroperitoneal lesions [2, 7]. It is important to mention that in regards to the current literature none of the recurrence incidents were observed in cases of omental lymphangiomas but rather in other intraperitoneal or retroperitoneal lymphangiomas.

## Conclusion

Omental lymphangiomas indeed represent a rare entity with equivocal clinical presentation. Therefore, the clinical suspicion of a pediatric surgeon has to be increased, especially when dealing with abdominal differential diagnostic approach. Regarding the efficacy of laparoscopy-assisted excision, we suggest that it represents an adequate and safe approach for the management of such lesions, providing that the possibility of complete resection is not compromised and that the procedure is carried out by experienced laparoscopy surgeons.
